# Effectiveness of vortioxetine in real-world clinical practice: Italian cohort results from the global RELIEVE study

**DOI:** 10.1192/j.eurpsy.2022.831

**Published:** 2022-09-01

**Authors:** S. De Filippis, A. Pugliese, K. Simonsen, H. Ren

**Affiliations:** 1 Villa Von Siebenthal, Psychiatry, Rome, Italy; 2 Lundbeck Italy, Medical Affairs, Milan, Italy; 3 H.Lundbeck A/S, Medical Affairs, Valby, Denmark

**Keywords:** Depression, real world evidence, vortioxetine, effectiveness

## Abstract

**Introduction:**

Major depressive disorder (MDD) is a debilitating disease in Italy affects 5.4% of people over 15 and 11.6% for the elderly. Efficacy of vortioxetine in adult patients with MDD was demonstrated in randomised controlled trials, there is a need for data on treatment in daily practice in Italy.

**Objectives:**

To present the effectiveness and safety data of vortioxetine in real-world setting from patients enrolled from Italy in the RELIEVE study.

**Methods:**

RELIEVE was a prospective, multi-national, observational study of outpatients initiating vortioxetine treatment for MDD at physician’s discretion. Data and outcomes of treatment of patients were collected at routine clinical visits. The primary outcome was functioning measured by SDS. Secondary outcomes included depressive symptoms measured by PHQ-9, cognitive funcion measured by PDQ-5, quality of life measured by EQ-5D-5L. Changes from baseline to month 6 were estimated with a linear mixed model of repeated measures approach.

**Results:**

A total of 231 patients (mean age, 55.5 years, 27.3% over 65 years, 62% female) were enrolled from Italy and included in the analysis. Mean(SD) SDS total score, PHQ-9, PDQ-5 scores at baseline were 17.8(7.58), 15.7(5.97) and 9.8(4.99), the scores(SE) decreased by 6.6(0.64), 5.9(0.47) and 3.6(0.36) from baseline to last visit. Mean(SE) EQ-5D-5L utility index increased by 0.13(0.01). Safety and tolerability profile of vortioxetine was in line with the established profile.

**Conclusions:**

Improvements in overall functioning, depressive symptoms, cognitive function and quality of life were observed in patients treated with vortioxetine, including a wide proportion of elderly patients in a real-world setting.

**Disclosure:**

A. Pugliese is an employee of Lundbeck Italy. K. Simonsen and H. Ren are employees of H. Lundbeck A/S.

